# Medicine

**Published:** 1902-03

**Authors:** Theodore Fisher


### Progress of the Medical Sciences.

### MEDICINE.

**Dr. Wentworth**[1] in a long paper refers to most of the recorded cases of **enlargement of the spleen associated with anæmia.** His survey of the subject is interesting, yet one can scarcely say that much fresh light is thrown upon the pathology of the disease. Perusal of the brief abstracts of the cases given brings out clearly the fact that the more fatal form of splenic anæmia is one of adult life. Of forty-two cases in which the ages are mentioned, only seven occurred under the age of twenty years, and only one under the age of ten. Anæmia associated with an enlarged spleen is comparatively common in infancy, but is not nearly so serious as the variety which occurs in later life. In the out-patient room it is not so very common to see the spleen diminish in size and the child's colour improve while under treatment, and later, one may sometimes have the opportunity of seeing the child attain apparently to perfect health. In connection with the blood affection of infancy, Dr. Wentworth takes some pains to show that there has been no ground for considering that this anæmia and **anæmia infantum pseudo-leukæmia** are two different diseases. The remarks he quotes of Fischl upon the examination of the blood in these cases are of interest. Fischl says: "After many years of work on the blood, one gradually acquires a certain resignation toward the results. The more one examines, the more uncertain one becomes of the significance of certain manifestations. This is especially the case in early childhood and infancy." This is an honest statement. Unfortunately, harm is often done to science by the reluctance of workers to admit that their labour has brought to light few positive facts. To return, however, to splenic anæmia. Stengel also lays little stress upon the appearances of the blood in the disease. He says: "It is quite evident from the study of the etiology, the

[1] *Boston M. & S. J.*, 1901, cxlv. 374 *et seq.*

pathological features, and the character of the blood, that anæmia infantum pseudo-leukæmia is not an independent disease; on the contrary, it seems to be a type of secondary anæmia, which may occur as a consequence of a variety of affections, and which owes its peculiar character of the blood to the age of the patient, rather than to the underlying disease." It is scarcely necessary to remark that the diseases most often associated with an enlarged spleen and anæmia in infants are rickets and syphilis. Wentworth mentions that Fischl in 1894 drew attention to the connection of this anæmia with the diseases, and he quotes the statistics of other writers who refer the association. Thus Fox and Ball found that rickets could not be excluded in a single case out of a series of sixty-three cases of enlargement of the spleen in children. Syphilis was also present in twenty-six cases, and probably in more. Marked anæmia, however, was not always present. This is a point worthy of note. Although enlargement of the spleen is not uncommon in rickets and in syphilis, the peculiar anæmia with the yellowish-brown tint of the skin occurs in only a small percentage of the cases. In splenic anæmia, the enlargement of the spleen may not necessarily be of great size. For example, on turning to my note-books, I find occurring within three months of one another, a spleen down to the iliac crest in a rickety child, aged fifteen months, without definite anæmia; and a spleen of the same size in a syphilitic child, aged eight months, also unassociated with any marked degree of anæmia; while in a child, aged 2¼ years, with the characteristic yellowish tinted skin of splenic anæmia, the spleen only descended one and a half inches below the costal margin. Although I estimated the amount of hæmoglobin in this case, I regret to say that a record of the result has not been entered in my notes; but I remember that the amount was very low. The notes, however, refer to the character of the blood corpuscles. In addition to the common appearance of a widespread deformity of the red blood-cells, an unusual number of megalocytes were present. Some were fully six times the size of an ordinary red blood-cell. It may be mentioned that there was no evidence of rickets or syphilis in this boy. Neither could I find any indication of these diseases in another boy, aged five, with splenic anæmia, who was first seen by me a year ago, and is at present in the hospital. Curiously enough, both of these boys had been born and brought up in localities which one would think are remarkably healthy. One boy has lived near the top of the Cotswolds, and the other high up on the Mendips. It would be a point possibly worthy of investigation whether those cases in which one cannot find evidence of rickets or syphilis are more chronic and more likely to eventually end fatally than in those in which these diseases are present.

Most of the cases of splenic anæmia met with in children occur under the age of two years. Wentworth quotes

Fede, who in sixty-four collected cases found only ten above the age of two years, and we probably may assume that cases which live usually get well before that age. I have not looked up the cases which have been under my care; but the steady improvement which has taken place in most of them would lead me to believe that both the anæmia and enlargement of the spleen would have entirely disappeared before the age of two years. The two boys mentioned above, however, if this be true, must be considered exceptions.

The relation of enlargement of the spleen to the anæmia is not clear. As already indicated, the size of the spleen is no indication of the degree of the anæmia. Dr. Wentworth enters somewhat fully into this point. He states that the spleen after death only shows fibrosis; and there is no evidence that fibrous tissue can produce toxins which injure the blood. This reasoning does not seem to warrant the conclusion that the disease may not arise in the spleen; that is to say, it does not necessarily militate against the view held by Banti that there is first an infection of the spleen, and that toxins are produced there. An infection which gives rise to fibrous tissue in the spleen may give rise also to toxins, which enter the circulation.

It is interesting to note that splenic anæmia in infants has been observed to affect several members of the same family; and comparatively recently Bovaird [1] and Brill [2] have recorded cases in which enlargement of the spleen associated with anæmia has occurred in more than one member of a family, at various ages. In Bovaird's two cases, the enlargement of the spleen apparently commenced at the age of three years. Three members of the family mentioned by Brill were affected, but only two came under his notice. One, a boy aged nine, died apparently of splenic anæmia, which, as in Bovaird's cases, commenced at the age of three; but the boy's brother and sister were not affected until they had reached the ages of eighteen and twenty-one. It may be mentioned in passing that six years ago a case of splenic anæmia occurring in a male patient, aged nineteen, under the care of Dr. Shaw, terminated fatally in the Bristol Royal Infirmary; and I understand that a brother of his is now dying from the same disease.

Dr. Parker, in the pages of this *Journal*,[3] has already commented upon the cases of Bovaird and Brill; but another brief reference may not be out of place. One characteristic of these cases appears to have been the very great size of the spleen; for example, in one of Brill's cases the enlargement is said to have given "the entire trunk the shape of a barrel." The heart was displaced upwards so that the apex was in the fifth interspace and the cardiac dulness as high as the first rib. In the other

[1] *Am. J. M. Sc.*, 1900, cxx. 377. [2] *Ibid.*, 1901, cxxi. 377.
[3] *Brist. M.-Chir. J.*, xix. p. 234.

case, the heart impulse was also displaced upwards to the third interspace, but the cardiac dulness reached only to the second rib. The enlargement of the abdomen, however, was very great. In both of Bovaird's cases also the spleen was of great size. Another feature was the chronicity. In three of the cases, the disease had lasted ten, eleven, and twelve years respectively; and the patients were living at the time the records were published. In the fourth case, after the disease had lasted thirteen years the spleen was removed; but the girl died three hours later. A fifth case, as has already been mentioned, ended fatally after the disease had lasted six years. The average length of life, it may be remarked, in splenic anæmia occurring in the adult is similar to that of pernicious anæmia, about two years. Bovaird has collected a few other cases from literature, which from the microscopic appearances of the spleen he considers similar to his own cases, but only in one instance was another member of the family affected.

**Acute Tuberculosis of the Spleen** is the subject of a short paper by D. D. Stewart; [1] and he mentions in passing that Bender has collected seven cases of enlargement of the spleen due to chronic tubercular affection of its substance. Only one case, however, of acute tuberculosis limited to the spleen had apparently been recorded. To this case Stewart adds another. A woman, aged twenty-nine, was seized with a sudden attack of fever, and died nine weeks later. At the autopsy the spleen was four times the normal size, and its surface studded with yellow tubercles the size of a pea. On section, about one half of the spleen had become transformed into a confluent tubercular mass, and tubercles of various sizes were scattered through the rest of the organ. Recent tubercles were also found in the kidneys, liver, and cerebral meninges, but the disease was obviously older in the spleen than elsewhere. Speaking from memory, without having paid special attention to the fact, one would be inclined to think that in cases of acute general tuberculosis the appearance of the tubercles on the spleen occasionally suggests that the disease has made some headway in that gland before the other organs of the body became affected. In one case I have seen miliary tuberculosis limited to the spleen and the liver, but this tuberculosis was not the cause of death. In a boy, aged five, who died of diphtheria, a caseous focus was found in one of the bronchial glands; but both of the lungs were free from disease. Recent tubercles were scattered over the surface of the spleen and throughout its substance, and tubercles were seen over the surface of the liver. The mesenteric glands showed no caseous foci, and there were no ulcers in the intestine. Apparently the inflammation of the bronchi, due to the presence of diphtheria, had caused dissemination of tubercle

[1] *Am. J. M. Sc.*, 1901, cxxii. 309.

bacilli contained in the caseous bronchial gland; and these bacilli had first attacked the spleen. Had the patient lived longer, the tuberculosis would no doubt have become general throughout the body.

THEODORE FISHER.

### SURGERY.

